# Charcot Neuroarthropathy Masquerading as Severe Acute Orthopedic Trauma: Severe Ramifications of Untreated Diabetes Mellitus

**DOI:** 10.7759/cureus.80526

**Published:** 2025-03-13

**Authors:** Max Slosarski, Ryan F Amidon, Christ Ordookhanian, Bilal Memon

**Affiliations:** 1 Medicine, Idaho College of Osteopathic Medicine, Eagle, USA; 2 Medicine, Medical College of Wisconsin, Milwaukee, USA; 3 Internal Medicine, University of California, Riverside School of Medicine, Riverside, USA

**Keywords:** charcot foot, charcot neuroarthropathy, diabetes mellitus, fracture, neuropathy

## Abstract

Charcot neuroarthropathy (CNA) is a progressively debilitating condition characterized by joint destruction and deformity due to neuropathy and mechanical trauma. It is most seen in patients with uncontrolled diabetes mellitus and resulting secondary peripheral neuropathy. Misdiagnosis is common, as the initial presentation of erythema, swelling, and warmth can mimic conditions such as cellulitis or osteomyelitis, and radiographic images may resemble that of severe trauma. Early recognition is crucial to prevent complications, including chronic deformity, ulceration, and amputation. In this case, a 42-year-old male patient with no documented medical history presented to the emergency department (ED) with worsening left ankle and foot pain after rolling his ankle on a flat surface. Imaging revealed acute, intra-articular fractures of the left hindfoot involving the talus, cuboid, navicular, and lateral cuneiform, with associated joint dislocations and severe soft tissue edema. These findings, initially concerning for severe limb trauma, in fact, represented that of CNA after underlying uncontrolled diabetes mellitus was diagnosed. While CNA is most commonly associated with diabetic peripheral neuropathy, it should not be excluded in the absence of a documented medical history of diabetes mellitus. Given the potential for misdiagnosis, early collaboration among specialists was essential in providing the patient with an accurate clinical determination. Early immobilization and offloading prevented further joint destruction, highlighting the critical role of early intervention in mitigating long-term complications such as chronic deformity or amputation.

## Introduction

Charcot neuroarthropathy (CNA) is a progressive, destructive condition of the bones, joints, and soft tissues of the foot and ankle, primarily associated with peripheral neuropathy. The pathophysiology involves an exaggerated inflammatory response to minor trauma or repetitive stress, resulting in osteolysis, joint subluxation, and eventual deformity [1]. The most common underlying etiology is diabetic peripheral neuropathy, although other causes, such as syphilis, trauma, and deformities, such as “claw toe” (metatarsal phalangeal joint hyperextension with interphalangeal flexion) or “hammer toe” (distal phalangeal extension), have been identified [2]. Conditions that should be considered in the differential diagnosis of CNA include osteoarthritis, septic arthritis, traumatic injury, cellulitis, osteomyelitis, gout, deep vein thrombosis, and heart failure. While the exact prevalence of CNA remains uncertain, it is estimated to affect up to 13% of individuals with diabetes mellitus and neuropathy during their lifetime [3]. 

Early detection of CNA is challenging due to its nonspecific presentation, which includes erythema, warmth, swelling, and varying degrees of pain. These signs often mimic other conditions, such as osteoarthritis, trauma, cellulitis, gout, or osteomyelitis, leading to frequent misdiagnoses and delays in appropriate treatment [4]. Imaging, particularly magnetic resonance imaging (MRI) and weight-bearing radiographs, is crucial in identifying early osseous and articular changes characteristic of CNA [5]. Without timely intervention, the condition progresses to chronic deformity, ulceration, and significantly increased risk of lower-extremity amputation [6].

Management of CNA depends on the stage of the disease, with initial treatment requiring immobilization and offloading during the acute phase to prevent further joint destruction. Once inflammation subsides, customized orthotic devices and surgical interventions may be necessary to restore function and prevent complications. Despite advances in understanding and treatment, CNA remains a diagnostic and therapeutic challenge, underscoring the importance of clinician awareness, early recognition, and a multidisciplinary approach to care [7].

## Case presentation

A 42-year-old male patient with no documented past medical history or family history presented to the emergency department (ED) with a chief concern of worsening left ankle and foot pain that began after rolling his ankle on a flat surface one week prior. The patient worked at a warehouse, frequently lifting heavy items. He continued to work with a limp despite the pain during this time. At the bedside, the pain was rated at four on 10, improving with a morphine injection. He was moderately hypertensive at 163/72 mmHg with other vitals within their reference ranges. He was afebrile at 37.1°C. No abnormalities were appreciated on cardiovascular, pulmonary, or eye exams. Notably, there was a severely swollen left ankle and foot that were tender to palpation, erythematous, and warm with a very limited range of motion. The swelling was visible to the left mid-calf, with ultrasound revealing edema but no deep vein thrombosis. Lab work yielded an elevated brain natriuretic peptide (BNP), C-reactive protein (CRP), erythrocyte sedimentation rate (ESR), and alkaline phosphatase (ALP), with a mildly elevated white blood count and a mild normocytic anemia (Table 1).

**Table 1:** Abnormal laboratory test results on emergency department admission Reference values are those expected for the patient’s age group and sex.

Laboratory test	Emergency department admission values	Reference values
White blood cell count	13.1 × 10^9^/L	3.4-9.6 × 10^9^/L
Hemoglobin	11.8 g/dL	13.2-16.6 g/dL
Alkaline phosphatase (ALP)	175 IU/L	44-147 IU/L
Brain natriuretic peptide (BNP)	1407 pg/mL	< 100 pg/mL
C-reactive protein (CRP)	6.14 mg/L	< 5 mg/L
Erythrocyte sedimentation rate (ESR)	71 mm/hour	0-15 mm/hour
Glucose (non-fasting)	255 mg/dL	70-140 mg/dL

The patient's creatinine level was relatively low at 0.5 mg/dL (reference range 0.74-1.35 mg/dL). Urinalysis revealed 2+ protein and 1+ glucose, otherwise unremarkable.

**Figure 1:**
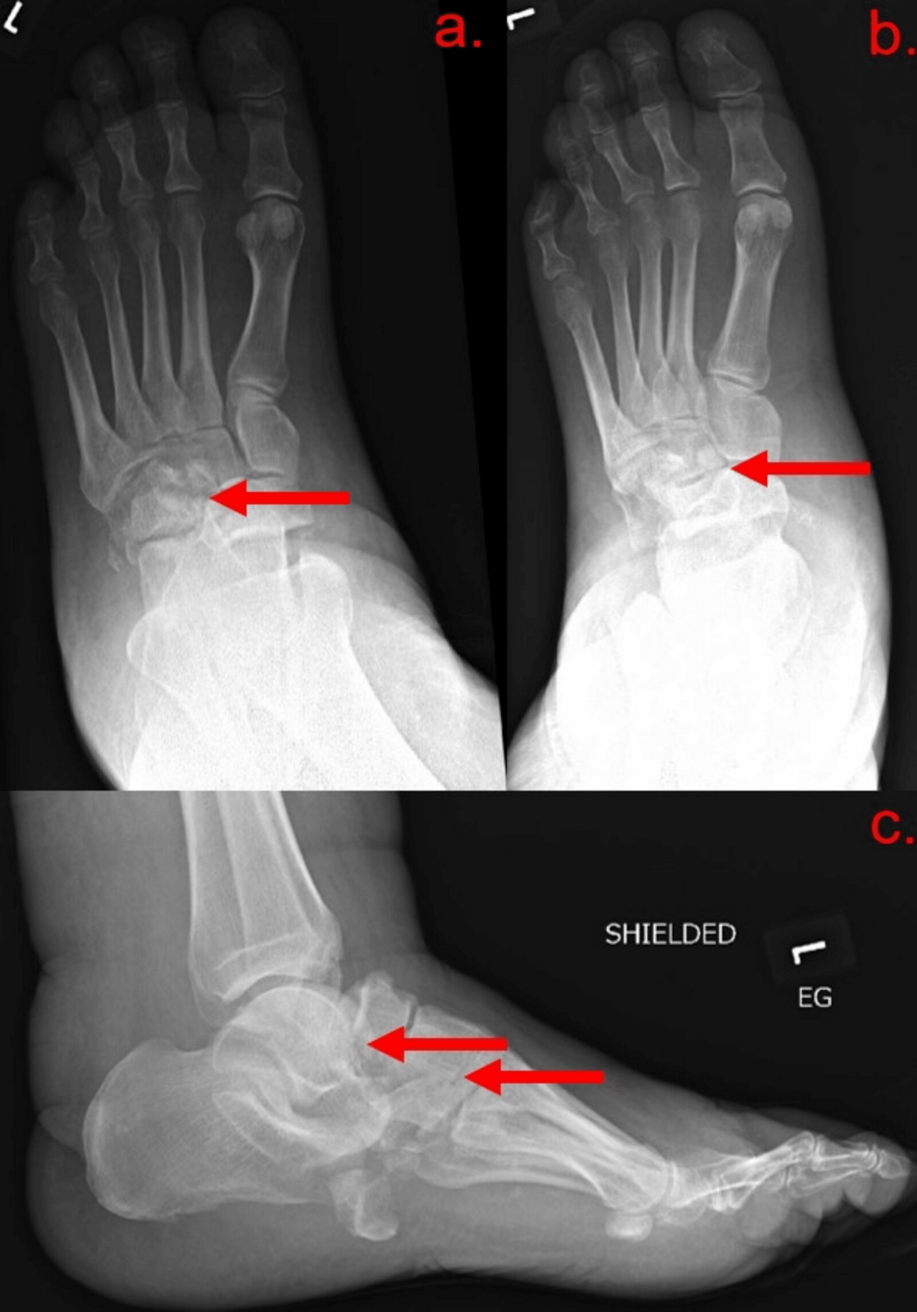
X-ray of the left foot in (a) anteroposterior (AP), (b) oblique, and (c) lateral views X-ray of the left foot in (a) AP, (b) oblique, and (c) lateral views, demonstrating extensive acute comminuted displaced intra-articular fractures of the left hindfoot with involvement of the talus, cuboid, navicular, and likely lateral cuneiform (red arrows). There is dislocation of the talocalcaneal and talonavicular joints. The left cuboid is significantly displaced. There is severe soft tissue edema.

At this point, the emergency team favored cellulitis or osteomyelitis based on the patient’s physical exam and lab results, with traumatic injury still high on the differential based on the patient's history. Vancomycin was started. The patient’s lack of documented medical history with elevated blood glucose led the team to order a glycohemoglobin test (HbA1c), which was elevated at 9.5%, diagnostic of diabetes mellitus. A radiograph of the left foot demonstrated extensive acute comminuted displaced intra-articular fractures of the hindfoot with multiple joint dislocations and severe soft tissue edema (Figure 1).

**Figure 2:**
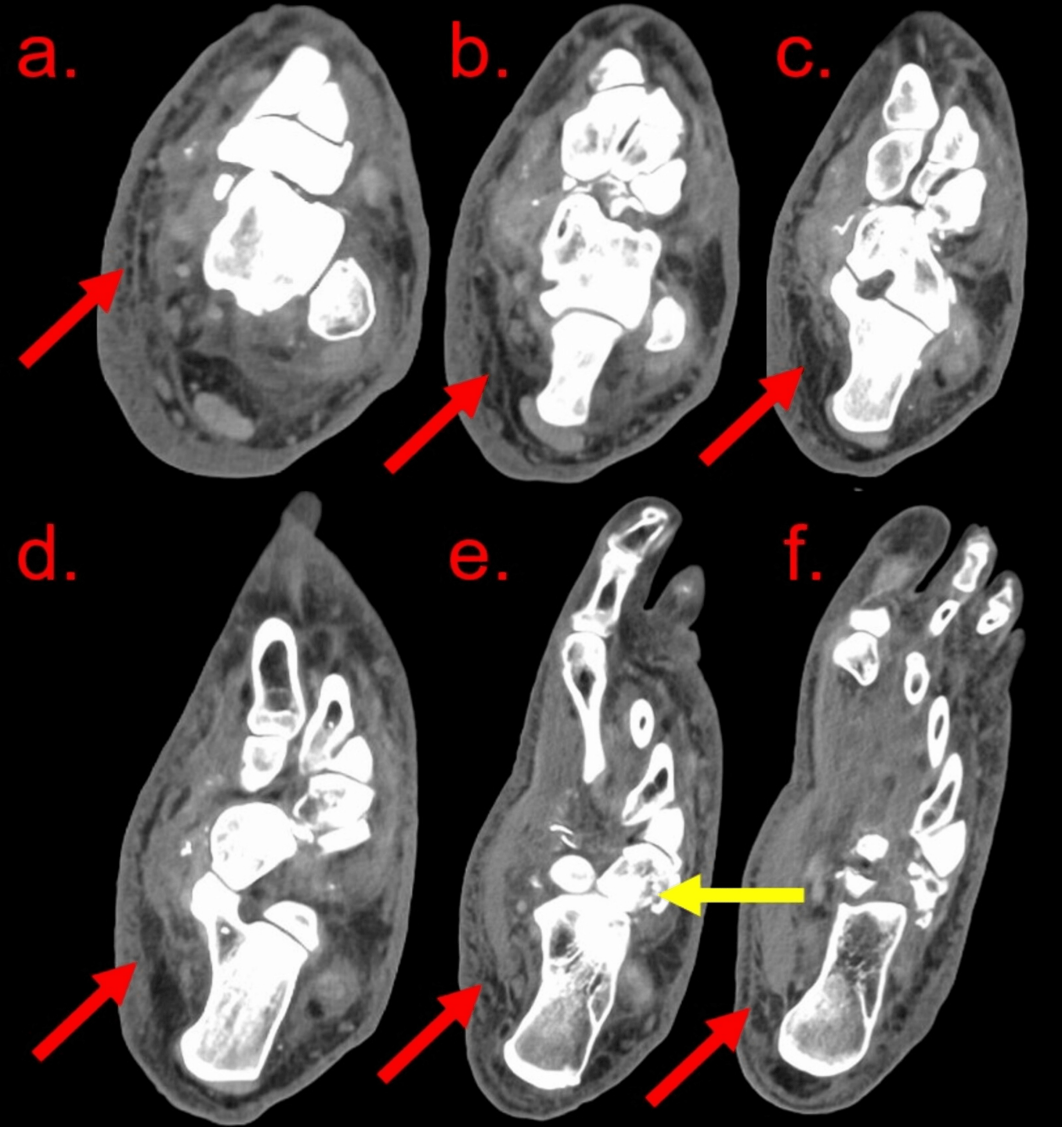
A CT scan without contrast of the left foot and ankle A CT scan without contrast of the left foot and ankle reveals diffuse subcutaneous fat stranding (red arrows) and edema throughout the ankle and foot. There is dorsal dislocation of the talonavicular joint and multiple small bony fragments along the superior and inferior margins of the navicular bone. There are markedly comminuted acute intra-articular fractures of the cuboid (yellow arrow) with disruption of the calcaneocuboid joint.

The orthopedic surgery team was consulted, recommending computed tomography (CT) imaging as the patient’s elevated blood sugar and recent trauma, in conjunction with the radiographic imaging, now was concerning for CNA or changes related to high-intensity trauma. The CT revealed diffuse fat stranding and edema throughout the left ankle and foot while redemonstrating the aforementioned fractures and dislocations (Figure 2).

The patient was diagnosed with CNA. He was started on diabetes medical management and was discharged for outpatient orthopedic and endocrinology follow-up. He was sent home with crutches for off-loading. One month later at follow-up, the patient’s HbA1c was reduced to 7.6% with medical management and dietary adjustments, and his left lower extremity was markedly less swollen and painful.

## Discussion

This case underscores the importance of maintaining a broad differential diagnosis in patients presenting with progressive lower extremity deformities, particularly in the context of minor trauma and persistent symptoms. While CNA is most associated with diabetic peripheral neuropathy, it should not be excluded solely based on the absence of a documented medical history [8]. Our patient presented with worsening left ankle and foot pain following an initial injury and continued weight-bearing activities. About 50% of patients with CNA can recall a traumatic event, and it can take as long as three weeks to observe CNA progression on plain radiographs [4]. Additionally, despite elevated inflammatory markers expected in CNA, a markedly elevated BNP, as seen in our patient, may lead to concern for heart failure as the primary cause of lower extremity edema. This was less concerning in our patient due to the unilateral nature of his lower extremity edema as well as unremarkable cardiovascular and pulmonary exam findings. However, while this should be further evaluated when seen in patients, elevated BNP is associated with diabetic peripheral neuropathy [9]. While the exact mechanisms are not fully understood, it is hypothesized that this is related to BNP’s peripheral vasodilation and anti-inflammatory effects. Given the complexity of the case and the potential for misdiagnosis, early collaboration among specialists was essential in providing the patient with an accurate clinical determination. Importantly, if the possibility of CNA as a diagnosis had not been pursued early, our patient would have likely been treated for cellulitis or osteomyelitis with broad-spectrum antibiotics, not only delaying proper treatment but also exposing the patient to iatrogenic complications.

Patients with CNA often have underlying diabetic neuropathy, the presence of which makes other microvascular complications such as diabetic nephropathy and retinopathy more likely [10]. Diabetic nephropathy is prevalent in patients with diabetic neuropathy due to shared pathophysiological mechanisms, including hyperglycemia-induced endothelial injury. Similarly, diabetic retinopathy risk is significantly increased in patients with existing diabetic complications, including neuropathy, nephropathy, and hypertension [11]. Interventions such as laser photocoagulation therapy and intravitreal injections of anti-vascular endothelial growth factor agents have been shown to mitigate its progression [12]. Overall, strategies such as intensive glycemic control, blood pressure regulation, and lifestyle modifications, including dietary adjustments and smoking cessation, can slow the development of diabetes complications. Recognizing and managing these comorbidities in patients with CNA is crucial in order to provide comprehensive care.

The timely multidisciplinary involvement in this case, including advanced imaging and input from orthopedic and radiology specialists, was instrumental in the accurate diagnosis of acute CNA [4]. Radiographic findings of comminuted intra-articular fractures and dislocations, combined with soft tissue edema and laboratory markers suggestive of systemic inflammation, directed the team toward the proper diagnosis. Early immobilization and offloading prevented further joint destruction, highlighting the critical role of early intervention in mitigating long-term complications such as chronic deformity or amputation [4]. This case demonstrates the necessity of a comprehensive approach to care, particularly in the event that a nondiabetic CNA may mimic cellulitis, osteomyelitis, or other more common conditions.

## Conclusions

Given the complexity of the case and the potential for misdiagnosis, early collaboration among specialists was essential in providing the patient with an accurate clinical determination. Importantly, if the possibility of CNA as a diagnosis was not pursued early despite the absence of known diabetes mellitus, our patient would have likely been treated for cellulitis or osteomyelitis with broad-spectrum antibiotics, not only delaying proper treatment but also exposing the patient to iatrogenic complications.

## References

[1] Jeffcoate WJ, Game F, Cavanagh PR (2005). The role of proinflammatory cytokines in the cause of neuropathic osteoarthropathy (acute Charcot foot) in diabetes. Lancet.

[2] Boulton AJ, Armstrong DG, Albert SF (2008). Comprehensive foot examination and risk assessment: a report of the task force of the foot care interest group of the American Diabetes Association, with endorsement by the American Association of Clinical Endocrinologists. Diabetes Care.

[3] Salini D, Harish K, Minnie P (2018). Prevalence of Charcot arthropathy in type 2 diabetes patients aged over 50 years with severe peripheral neuropathy: a retrospective study in a tertiary care South Indian hospital. Indian J Endocrinol Metab.

[4] Madan SS, Pai DR (2013). Charcot neuroarthropathy of the foot and ankle. Orthop Surg.

[5] Rosskopf AB, Loupatatzis C, Pfirrmann CW, Böni T, Berli MC (2019). The Charcot foot: a pictorial review. Insights Imaging.

[6] Berli M, Vlachopoulos L, Leupi S, Böni T, Baltin C (2017). Treatment of Charcot neuroarthropathy and osteomyelitis of the same foot: a retrospective cohort study. BMC Musculoskelet Disord.

[7] Vopat ML, Nentwig MJ, Chong AC, Agan JL, Shields NN, Yang SY (2018). Initial diagnosis and management for acute Charcot neuroarthropathy. Kans J Med.

[8] Milne TE, Rogers JR, Kinnear EM, Martin HV, Lazzarini PA, Quinton TR, Boyle FM (2013). Developing an evidence-based clinical pathway for the assessment, diagnosis and management of acute Charcot neuro-arthropathy: a systematic review. J Foot Ankle Res.

[9] Yan P, Wan Q, Zhang Z, Xu Y, Miao Y, Chen P, Gao C (2020). Association between circulating B-type natriuretic peptide and diabetic peripheral neuropathy: a cross-sectional study of a Chinese type 2 diabetic population. J Diabetes Res.

[10] Jansen RB, Holstein PE, Jørgensen B, Møller KK, Svendsen OL (2021). Risk factors for development of nephropathy in patients with a diabetic Charcot foot. BMC Res Notes.

[11] Al-Rubeaan K, Abu El-Asrar AM, Youssef AM (2015). Diabetic retinopathy and its risk factors in a society with a type 2 diabetes epidemic: a Saudi National Diabetes Registry-based study. Acta Ophthalmol.

[12] Mansour SE, Browning DJ, Wong K, Flynn HW Jr, Bhavsar AR (2020). The evolving treatment of diabetic retinopathy. Clin Ophthalmol.

